# On the Use of Soda

**Published:** 1803-03-01

**Authors:** R. Simmons

**Affiliations:** Oxendon Street, Leicester Square


### *On the* USE *of* SODA; *communicated by* MR. R. SIMMONS, *of Oxendon Street, Leicester Square.*

THE subject of the remedy alluded to, Mary Frazer, a florid, healthy-looking young woman, aged 19, was admitted into St. Martin's Parish Infirmary in April last, for the cure of a swelling in the right groin. At the time of her admission, it was hard and painful, and it went on to a state of suppuration and broke of itself. The sore in the groin, instead of healing kindly, which might naturally have been expected from her general appearance of health, soon put on an ill-conditioned aspect, and became extremely irritable, spreading wide and ulcerating very deep, giving that appearance usually denominated phagedenic. From her situation in life, the case was considered as venereal, though there did not exist any other venereal symptom, and she was put on a course of mercurial friction; but getting worse under it, the most usual remedies in such cases were had recourse to, and each persevered in as long as any kind of benefit was to be expected from their use; viz. bark, opium, cicuta, sarsaparilla, the nitrous acid, with carrot and hemlock poultices, poppy fomentations, sea water, and other remedies esteemed useful in phagadænia; but she continued getting worse, until her health became much impaired, and her appetite was nearly gone.

Not knowing, at this time, any particular remedy that could be depended on, so as to produce a cure, I determined on trying the effects of soda, and thought that the common crystals would answer for that purpose; which I used both externally and internally, and its good effects were soon very striking. The sore, which had hitherto preserved an ugly, gleeting, ill-conditioned appearance, put on a healthy granulating surface; her appetite mended and her general health speedily improved; and by continuing the use of this remedy, she is now nearly well, the sore being too trifling to mention. The mode adopted for making use of this remedy, was to dissolve an ounce of the crystals of soda in a quart of water, and to apply it externally in the form of a poultice with crumbs of bread; at the same time, taking three table-spoons full of this solution three times a day.

*February* 8, 1803.

